# Dopamine Signaling Regulates Fat Content through β-Oxidation in *Caenorhabditis elegans*

**DOI:** 10.1371/journal.pone.0085874

**Published:** 2014-01-22

**Authors:** Alexandre Guimarães de Almeida Barros, Jessika Cristina Bridi, Bruno Rezende de Souza, Célio de Castro Júnior, Karen Cecília de Lima Torres, Leandro Malard, Ado Jorio, Débora Marques de Miranda, Kaveh Ashrafi, Marco Aurélio Romano-Silva

**Affiliations:** 1 Instituto Nacional de Ciência e Tecnologia de Medicina Molecular, Faculdade de Medicina da Universidade Federal de Minas Gerais, Belo Horizonte, Minas Gerais, Brazil; 2 Departamento de Fisiologia e Biofísica, Instituto de Ciências Biológicas da Universidade Federal de Minas Gerais, Belo Horizonte, Minas Gerais, Brazil; 3 Departamento de Física, Instituto de Ciências Exatas da Universidade Federal de Minas Gerais, Belo Horizonte, Minas Gerais, Brazil; 4 Department of Physiology, University of California San Francisco, San Francisco, California, United States; Virginia Commonwealth University, United States of America

## Abstract

The regulation of energy balance involves an intricate interplay between neural mechanisms that respond to internal and external cues of energy demand and food availability. Compelling data have implicated the neurotransmitter dopamine as an important part of body weight regulation. However, the precise mechanisms through which dopamine regulates energy homeostasis remain poorly understood. Here, we investigate mechanisms through which dopamine modulates energy storage. We showed that dopamine signaling regulates fat reservoirs in *Caenorhabditis elegans*. We found that the fat reducing effects of dopamine were dependent on dopaminergic receptors and a set of fat oxidation enzymes. Our findings reveal an ancient role for dopaminergic regulation of fat and suggest that dopamine signaling elicits this outcome through cascades that ultimately mobilize peripheral fat depots.

## Introduction

Obesity has emerged as a major health problem that is implicated in several human diseases [1]. Studies aimed at understanding the physiological mechanisms of body weight regulation have revealed that the central nervous system [2] plays a critical role in energy homeostasis. The CNS integrates internal and external cues of energy demand and availability to coordinate behavioral, physiological, and metabolic responses, thereby keeping energy stores stable over time [3] [4]. However, the neural circuits and molecular mechanisms that underlie the perception of and responses to different energetic states remain poorly understood.

A number of findings point to the neurotransmitter dopamine as a central regulator of energy balance. Knock-out mice lacking dopamine exhibit dramatically reduced food intake and die of starvation a few weeks after birth [5] [6]. Leptin, insulin and ghrelin, key hormones that communicate the energetic status of the periphery to the CNS, act on dopaminergic neurons [7]. For instance, leptin signaling triggers production of dopamine in a subset of neurons and intact dopamine signaling is required for manifestations of hyperphagia and weight gain in leptin deficient ob/ob mice [8] [9] [10]. Based on these studies, the function of dopamine signaling in the regulation of energy balance has been ascribed to this neurotransmitter's role in motivating food-seeking behavior. Human studies, however, suggest a more complicated function. Compared to lean individuals, levels of the dopamine D2 receptors have been reported to be reduced in obese persons and a specific dopamine D2 receptor polymorphism has been linked to the development of obesity [11] [12]. Thus, while various findings highlight a role for dopamine signaling on energy balance, the precise relationship of dopamine and obesity remains unresolved.

*Caenorhabditis elegans* provides a genetically tractable system for rapid and functional analysis of fat regulatory pathways in the context of an intact animal [13] [14]. As in mammals, *C. elegans* neural sensory mechanisms gauge environmental conditions and coordinate myriad behavioral and physiological responses [15] [16]. Thus far, the effects of dopamine signaling on energy related pathways in *C. elegans* have been best characterized in the context of food-seeking behaviors. In hermaphroditic worms dopamine production and release are confined to the ADE, PDE and CEP neurons, which are primarily mechanosensory [17] [18] [19]. Physical contact with irregular surfaces, such as those corresponding to bacteria, causes dopamine's release leading to worms reduced movement [20] [17]. Dopamine signaling also increases the frequency of the worm's turning events [21]. Both the slowed movement rate and the enhanced turning frequency have been postulated as a mechanism for maximizing the time that foraging animals spend in the vicinity of a food source [20] [21] [17]. Moreover, studies have shown that dopamine signaling is required for adaptation to odorants, a process that could modify *C. elegans* responses to food cues [22] [23].

In this study we used the nematode *C*. elegans to investigate the effects of dopamine signaling on fat content. To this end, we measured fat reservoirs of animals exposed to different concentrations of dopamine and carried out genetic analyses of potential molecular targets implicated by fat deposition.

## Materials and Methods

### *C. elegans* maintenance

Nematodes were maintained using standard procedures as described previously [24]. Wild type reference strain was N2 Bristol. Some strains were provided by the CGC, which is funded by NIH Office of Research Infrastructure Programs (P40 OD010440). CF2808 and CF2805 were kindly provided by the Kenyon Lab. The following strains were used: LX636 *dop-1(vs101)*, LX702 *dop-2(vs105)*, LX703 *dop-3(vs106)*, RB1254 *dop-4(ok1321)*, CB1112 *cat-2(e1112)*, LC33 *bas-1(tm351)*, RM2702 *dat-1(ok157)*, LX706 *dop-1(vs100);dop-2(vs105)*, LX705 *dop-1(vs100);dop-3(vs106)*, CF2808 *dop-1(vs100);dop-4(ok1321)*, LX704 *dop-2(vs105);dop-3(vs106)*, LX734 *dop-1(vs100);dop-2(vs105);dop-3(vs106)*, CF2805 *dop-1(vs100);dop-2(vs105);dop-3(vs106);dop-4(ok1321)*, RB1700 *f59f4.1*(*ok2119*), MT9668 *mod-1(ok103)*, VS24 *kat-1(tm1037)*, VS20 *hjIs67*[*atgl-1p::atgl-1::GFP*]. All strains were outcrossed at least 4×. Strains were synchronized by hypochlorite treatment of gravid adults. Eggs were allowed to hatch overnight in M9 and used next morning. For all experiments strains were kept at 20°C.

### Dopamine treatment

Dopamine hydrochloride (Sigma, Germany) was freshly dissolved in 0.1 M HCl and added to the top of NGM plates seeded with either OP50 or RNAi clone (HT115) to the desired concentration. For vehicle-control plates, a 0.1 M HCl solution was used. For experiments, L4-stage worms were transferred to vehicle or dopamine containing plates and the desired parameter measured 24 hours later unless otherwise noted. For biochemical and fatty acid oxidation assays L3-stage animals were used to avoid interference of yolk from eggs.

### Pharyngeal pumping rate assay

Pharyngeal pumping was measured in healthy animals on food at room temperature. The number of contractions of the pharyngeal bulb was counted over 10 s, in triplicate, and the average multiplied by 6 to give pumps/min. All experiments were repeated at least twice.

### Locomotory rate assay

Animals were transferred one by one to fresh plates seeded with 100 µL of OP50. After a 10 minute adaptation period, 1 minute digital videos were captured at ∼12 frames/s using a dissecting microscope outfitted with a CCD camera. Worm velocity was measured by the “worm tracking” function of the Nikon analysis software. Experiments were performed at room temperature and repeated twice.

### Defecation rate assay

Animals on food from vehicle or dopamine treatment plates were monitored. For each worm an average of three intervals between two defecation cycles was measured. The defecation cycle was identified as a peristaltic contraction beginning at the posterior body of the animal and propagating anteriorly followed by feces expulsion. Experiments were done at room temperature and repeated at least twice.

### Progeny production assay (number of eggs per worm and eggs released over time)

Well-fed animals from vehicle or dopamine treatment plates were used. To measure the number of eggs inside the uterus, individual worms were lysed using a bleach solution and the number of eggs released was counted. To measure the egg-laying rate, individual worms were transferred to fresh plates and allowed to lay eggs for one hour. The next day the number of progeny, including crawling worms and dead eggs, on each plate was counted. Noteworthy, the number of dead eggs was negligible. Experiments were performed at room temperature and repeated at least twice.

### Vital Dyes lipid staining

Standard Nile Red and BODIPY lipid staining were conducted as described previously with minor modifications [13]. Briefly, Nile Red and BODIPY stock solutions were dissolved in M9 and added to the top of NGM plates at final concentrations of 50 nM and 0.02 µg/mL, respectively. For acute Nile Red staining, a solution 100× concentrated was spread over animals on food. After one hour, worms were collected and mounted on agarose pads for microscopy.

### Sudan Black and Oil-Red-O and LipidTOX lipid stainings

Fixative-based stainings were performed as described previously [25]. Briefly, worms were collected and fixed with 2% paraformaldehyde. After a permeabilization step with DTT, animals were prepared according to each protocol. For LipidTOX, permeabilized animals were exposed to 1× LipidTOX in 1× PBS. For Oil-Red-O, animals were dehydrated in 60% isopropyl alcohol and incubated with 60% Oil-Red-O solution. For Sudan Black, worms were dehydrated in an ethanol series and incubated with 75% ethanol saturated Sudan Black solution. All incubations were performed overnight.

### Images acquisition

For standard Nile Red and BODIPY, fluorescent images were acquired on a Zeiss Axioplan II inverted microscope, outfitted with a digital CCD camera, using identical settings, suitable fluorescence filters and the same exposure times. For LipidTOX and acute Nile Red, a Leica TCS SP5 confocal microscope fitted with an argon excitation laser was used. Images were acquired using identical settings including PMT's reading spectrum and gain. Images used for quantification had pixel intensities below saturation levels. The fluorescence in the first half of the distance between the beginning of the intestine and the vulva was quantified. Usually this region encompasses the first two to three segments of intestinal cells. For each condition or genotype worms were randomly selected under a dissection microscope and mounted for analysis. Each experiment was repeated at least twice.

### Feeding RNAi

RNAi feeding on agar plates was performed as described previously [26]. Briefly, plates containing IPTG and proper antibiotic were seeded with *E. coli* strain HT115 carrying each of the selected RNAi clones. Plates were induced overnight and used the next day. Dopamine and Nile Red were added on top of each plate and allowed to equilibrate overnight. Plasmid L4440 without inserts was used as a control. Synchronized L1 animals were transferred to plates containing RNAi/bacteria. They were fed on RNAi plates throughout their growth and treatment. The identity of each RNAi clone was blinded during experiments. Knockdowns that resulted in a suppression of the dopamine induced fat phenotype of more than 80% of the control animal's reduction were considered for further analyses. For the metabolic screen, data were acquired with Acumen (TTP LABTECH, England) a microplate fluorescence citometer, and double checked in a visual-based assay. For missing values or values with SD bigger than 20% from the automatic assay, only the visual-based assay data were considered. Data from both assays had the same trend with the former being more sensitive for variations in intensity.

### CARS Imaging

For the coherent anti-stokes Raman imaging [27], we used a picosecond optical parametric oscillator (picoEmerald - APE) with the stokes tuned at 1064 nm and pump at 817 nm in order to match the lipid CH2 stretching mode at 2845 cm^−1^ [28] [29]. Both laser pulses are synchronized in time and space and directed to a galvanometric mirror imaging microscope (Nikon Ti-U inverted microscope coupled to a TriM Scope II - LaVision BioTec system) and focused onto the sample by a 10X Nikon objective. The backscattered signal is then directed to a set of dichroic and band pass filters in order to remove the pump and stokes lasers and to detect only the anti-stokes signal in a photomultiplier (Hamamatsu H7422-40).

### Lipid extraction and Triglyceride measurement by Thin-Layer Chromatography (TLC)

10,000 late L4-stage animals were collected. Total lipids were extracted at room temperature as described previously [30]. Based on the amount of protein, normalized volumes from each sample were loaded on silica gel 60 plates (EMD CHEMICALS, Germany) and developed using the solvent system hexane:diethyl ether:acetic acid (70/30/1). Known quantities of lipid standards (NUCHECK, CA United States) were loaded alongside samples for generation of a standard curve. Phosphomolybdic acid (SIGMA-ALDRICH, St. Louis, United States) was used for revealing TLC plates. Plates were scanned (300 dpi) for densitometry. Measurements were performed in duplicate and absolute quantities calculated based on standard curve densitometry from the same plate.

### Images quantification and densitometry

Image quantification and densitometry were performed using ImageJ software (NIH).

### Fat acid oxidation assay

Worms were exposed to dopamine from L3 to late L4 larval stages (24 hours) as described previously. The animals were collected and washed three times with S-Basal to remove bacteria. For each sample, a BSA-oleic acid solution (0.5 mM cold oleic acid/65 mCi/L hot oleic acid/0.55 mM NaOH/8.25 g/L BSA) was added sequentially. Samples were incubated for 60 minutes at room temperature in contact with hot oleic acid solution (BSA-oleic acid solution). This interval was sufficient to allow no dopamine and dopamine exposed worms to absorb and metabolize radiolabeled oleic acid. Subsequently, trichloroacetic acid (10% w/v) was used to stop the reaction. After spin down, supernatants were transferred to new tubes and 1× PBS and 5 M NaOH added in a proportion of 0.1/0.25, respectively. Samples were loaded onto Dowex resin (SIGMA-ALDRICH 44340 - 1×8 200–400 MESH Cl, St. Louis, United States) columns and eluents collected into scintillation vials. Scintillation fluid was added to each vial and radioactivity was measured. Protein content was used to normalize radioactivity. Experiment was repeated at least twice.

### qPCR

Gene expression measurement through qPCR was performed as describes previously [31] [32] [33]. Briefly, three independent populations of 30,000 late L4-stage animals were collected and washed three times with S-Basal to remove bacteria. They were frozen in liquid nitrogen and processed next day. For RNA extraction, worm were thawed at 65°C for 10 minutes and RNA was isolated using Trizol (Invitrogen). Isolated total RNA was treated with DNAse and purified using RNAeasy (Qiagen) according to the manufacture's protocol. cDNA was prepared using SuperScript III First-Strand Synthesis (Invitrogen). For PCR reactions we used PlatinumTaq (Invitrogen) and amplicon formation was monitored using SYBR-Green (Invitrogen). All PCR reactions and fluorescence collection were carried out in an ABI-7500 real-time PCR machine (ABI). Reactions were run in triplicates and samples from three different populations were used for comparisons.

Differences in genes expression were assessed through the comparative Ct method that uses the following equation: 2^−Δ Ct^, where −Δ Ct = − (Ct dopamine treated or control animals − Ct geometric mean of housekeeping genes of the same sample) and Ct =  cycle threshold calculated by the qPCR software (ABI 7500). 2^−Δ Ct dopamine^/2^−Δ Ct no dopamine (control)^ ratio indicates fold changes in gene expression after exposure to dopamine. qPCR data were normalized by using a set of control genes previously reported [31]. Ct values and primer sequences available in Table S1.

### Statistical Analyses

Student's t-test or one-way Analysis of Variance (ANOVA) followed by Bonferroni post-test was used to check for significant differences between means.

## Results

### Dopamine signaling modulates fat content

**Figure 1:**
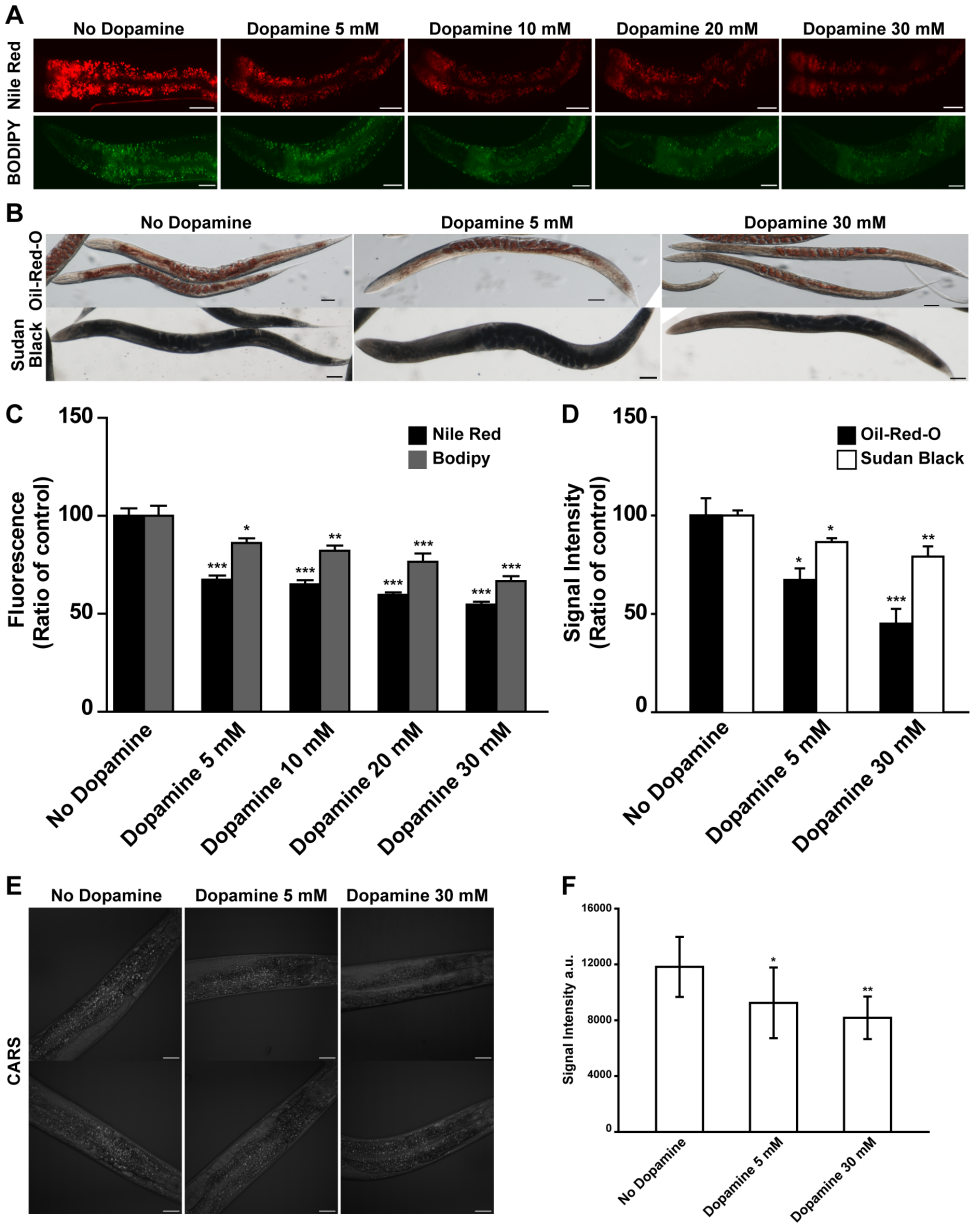
Dopamine reduces fat content of wild type animals. (A–D) Animals exposed to increasing concentrations of dopamine exhibit reduced fat stores as visualized by vital dyes Nile Red and BODIPY-labeled fatty acids, fixative-based dyes Oil-Red-O and Sudan Black and CARS microscopy. (A) Representative images and (C) quantitation of fluorescence intensities. Black and gray bars represent Nile Red and BODIPY respectively. (B) Illustrative images and (D) quantitation of stain intensities. Black and white bars represent Oil-Red-O and Sudan Black respectively. (E) Representative images and (F) quantification of CARS signal intensities. (C–D) Data are showed as percentage of control (“No dopamine”) average ± SEM. (n = 12 to 15 animals per condition for Nile Red and BODIPY. n = 5 to 8 animals per condition for Oil-Red-O and Sudan Black). (F) Data are showed as absolute intensity ± SD. (n = 8 to 10 worms per condition). * p<0.05, ** p<0.01, *** p<0.001 compared to “No dopamine” animals.

To explore whether dopamine signaling modulates worm's fat reservoirs several methodologies were used to assess lipid content. First animals were exposed to different concentrations of exogenous supplied dopamine and stained with vital dyes Nile Red and BODIPY-labeled fatty acid. We found that exposure of *C. elegans* to exogenous dopamine reduced fluorescence signals (Figure 1 A and C, Figure S2 A). This effect was dose-dependent and reversible upon removal of drug treatment (Figure S1 A and B, Figure S2 B). In addition, the dopamine concentration range employed was the same that has been widely used to investigate properties of dopamine signaling in *C. elegans* [34] [22] [23] [20]. However, vital dyes are not the gold standard for fat measurement anymore. Although they have been used extensively to investigate fat content in multiple species and examination of various evolutionarily conserved fat pathways in *C. elegans* results in expected Nile Red phenotypes [13] [35] [31] [36], several studies have recently called into question the validity of these dyes for analyzing *C. elegans* fat [37] [38]. To address this question and further explore dopamine's effects on fat deposition Sudan Black and Oil-Red-O staining procedures were used. Both are fixative based dyes and are supposed to not interact with xenobiotic clearance pathways. The results obtained with Sudan Black and Oil Red O were similar to those observed with vital dyes but the measurable decreases were smaller (Figure 1 B and D).

Worm's fat reservoirs are dynamic and tightly regulated. Lipid droplets have been observed within different compartments including the intestine, hypodermal cells, and gonads [39]. Nile Red, used according to standard protocol, outlines specifically intestinal cells structures while Bodipy stains other sites as well. Dye structures are different and may interfere with their permeability and handling by the detoxification system. Studies have suggested that variations in standard staining protocols may broaden the spectrum of marked structures. For instance, use of fixative approaches for Nile Red and Bodipy and a change in staining time for Bodipy highlight fat droplets in a pattern similar to Sudan Black and Oil-Red-O. To explore whether a variation in worm's contact to Nile Red improves fat staining a one-hour exposure timeframe was adopted. Acute exposure allowed observation of lipid droplets at different places beyond intestinal cells (Figure S3). Moreover, marked structures co-localized with ATGL, a lipase associated with lipid droplets [40]. Therefore, it is possible that the difference in the signal magnitude observed when Nile Red is used compared to other dyes may represent specific properties of the assays. Furthermore, fat measurement methodologies using bulk worm extracts merge lipid droplets from diverse tissues and compartments, while fixative procedures may modify internal medium. These may limit interpretation under some circumstances. For instance, considering the great number of worms necessary for biochemical assays, it is possible that lipid variations inside populations may bring misleading results. To observe whether lipids fluctuate within populations, extracts form worms in different larval stages were evaluated through thin layer chromatography (Figure S4). As expected worms in different stages showed different lipid content. Noteworthy, different extracts from worm's populations at the same developmental stage displayed variable lipid content. We hypothesized that this is because developmental stages have a gaussian's distribution pattern in large worm populations. This means that, at the same time, part of the worms have more advanced developmental stage in comparison with other part of the population. Therefore, one benefit in adopting microscopy approaches to quantify fat is that they allow morphological classification of worms being compared. Studies have shown that the use of CARS microscopy for fat measurement is feasible and evades possible dye staining protocol biases [28] [29]. Therefore to verify our results obtained from dye staining trials, CARS microscopy was used. Worms exposed to exogenous dopamine have a reduced amount of triglycerides as measured by CARS signal (Figure 1 E and F). The observed correlations between Nile Red, Bodipy, Sudan Black, Oil-Red-O and CARS support the validity of vital dyes for assessing *C. elegans* fat however vital dyes should not be used in isolation. It is indicative that inherent differences in sensitivities of different methods likely account for some of the previously noted discrepancies. For further experiments Nile Red was adopted as a probe for fat measurement because of its technical straightforwardness and feasibility to high throughput screenings. Relevant results were confirmed with fixative based assays.

### Dopamine modulation of *C. elegans* fat levels depends partially on the GPCR family of dopamine receptors

**Figure 2:**
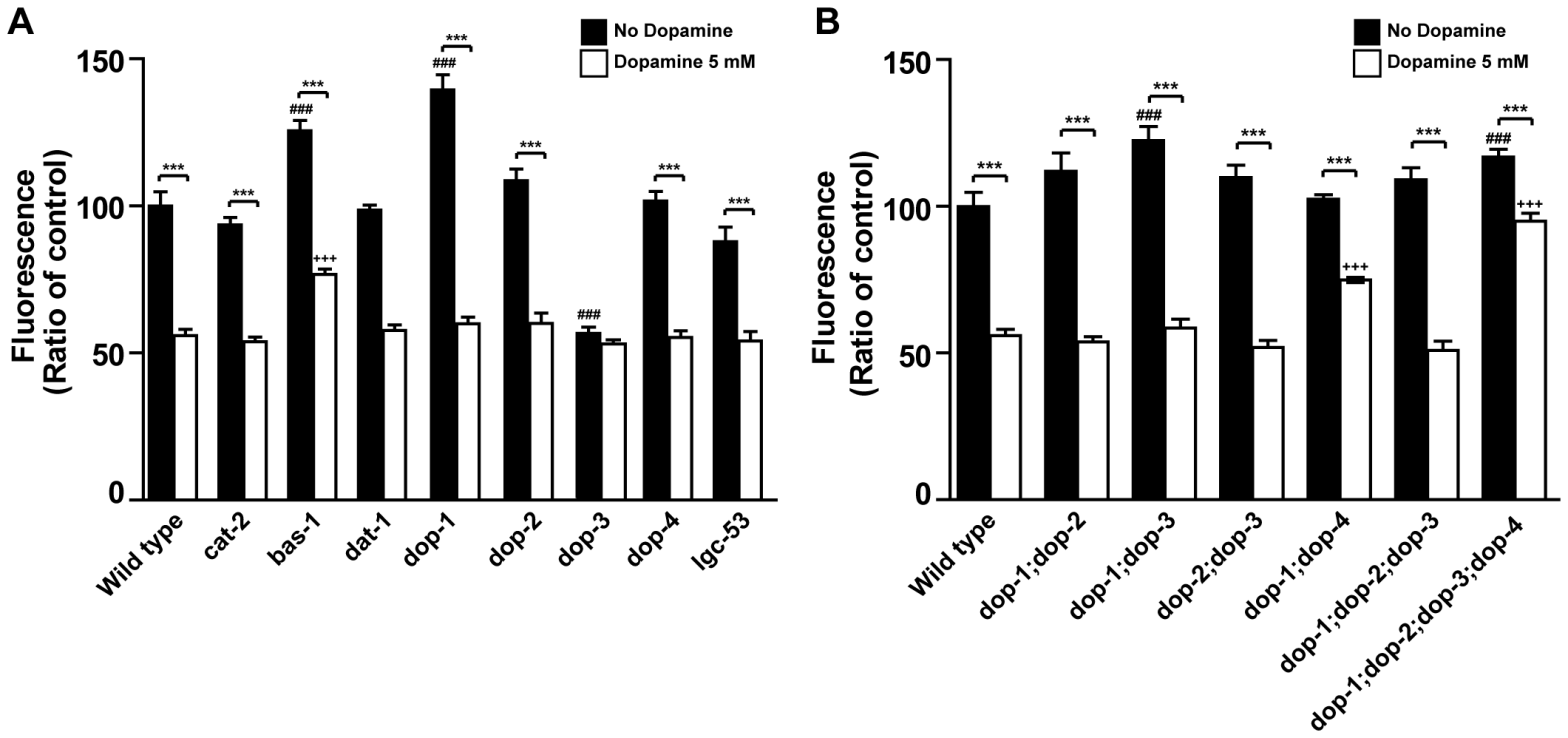
Dopamine signaling effects on fat depends on dopamine receptors. (A–B) Fat levels of various mutants with or without exposure to dopamine. Black and white bars represent vehicle and 5 mM dopamine treated animals, respectively. Data are shown as percentage of wild type vehicle-treated average ± SEM. (n = 10–20 animals per condition per genotype). *** p<0.001 compared to vehicle-treated animals of the same genotype, ### p<0.001 compared to vehicle-treated wild type animals, +++ p<0.001 compared to dopamine-treated wild type animals.

*C. elegans* synthesize dopamine from tyrosine in a sequence of two reactions catalyzed by tyrosine hydroxylase (CAT-2) and aromatic amino acid decarboxylase (BAS-1) [41] and clear it in part through capture by dopamine transporter (DAT-1) [42]. To evaluate whether the endogenous dopaminergic system modulates lipids content we first characterized the fat phenotypes of *cat-2*, *bas-1* and *dat-1* mutants (Figure 2 A). As expected, loss of *bas-1* increased Nile Red fluorescence levels, however *cat-2* and *dat-1* mutants' phenotypes were indistinguishable from wild type control animals. Previous studies have found that *cat-2* mutants are not completely dopamine deficient and that dopamine operates extra-synapticaly in *C. elegans* [20] [43] [44]. It is possible that low levels of dopamine may be sufficient to maintain wild type fat levels and dopamine's clearance may require pre-synaptic transporter capture as well as enzymatic degradation for ending dopamine neurotransmission. Therefore dopamine levels in *dat-1* mutants may not be sufficient to cause a measurable reduction in fat storage, while dopamine levels in *cat-2* mutants may be sufficient to maintain normal fat levels.

Furthermore, while the *bas-1* mutant phenotype suggested that loss of endogenous dopamine synthesis may cause fat accumulation, interpretation of its results was complicated by the fact that BAS-1 is also required for serotonin production and serotonin deficient animals have been reported to have a high fat phenotype [15] [45]. To shed light on this question, wild type animals were exposed to each neurotransmitter. Treatment with both amines alone reduced Nile Red fluorescence and showed a trend to further reduction when in combination although not significantly (Figure S1 C). This suggests that dopamine and serotonin may operate in parallel to modulate fat even though this does not exclude possible crosstalk between them. Serotonin reduces fat through activation of *ser-6* and *mod-1*, a GPCR and a serotonin-gated chloride channel respectively. To further check for dopamine's potential unspecific actions, *mod-1* loss-of-function mutants were exposed to different concentrations of dopamine. Dopamine treatment reduced *mod-1* fat content by the same degree as wild-type control animals (Figure S1 D). Studies suggest that serotonin elicits transcriptional upregulation of several metabolic genes to reduce fat reservoirs [15]. To shed light whether dopamine operates nonspecifically through serotonin pathways, transcriptional levels of serotonin regulated metabolic genes were assessed by quantitative real-time polymerase chain reaction (qPCR). Dopamine exposure had no effect over these genes expression levels (Figure S5). Our results thus suggest that dopamine has a specific role in fat reservoirs control.

The human genome codes for two classes of dopamine receptors [46]. Based on sequence similarities, pharmacological profiles, and biochemical properties at least five dopamine receptors have been validated in *C. elegans* to date. Four of them, DOP-1, DOP-2, DOP-3 and DOP-4, are seven transmembrane G protein coupled receptors, while one, LGC-53, is a ligand-gated chloride channel [43] [20] [47] [48] [49]. As in humans, worm receptors can be grouped into two families with opposite functions. We hypothesized that chronic exposure to exogenous dopamine may favor one group of receptors. This may happen by intrinsic differences in desensitization mechanisms between them. In this scenario chronic activation could unbalance dopamine's signaling. To evaluate whether unbalance of the endogenous dopamine system modulates fat levels, Nile Red fluorescence was assessed in animals carrying single or combination loss-of-function mutations in dopaminergic receptors (Figure 2 A and B). Of the five receptors tested only *dop-1* mutants had increased fat levels. In contrast *dop-3* mutants had decreased fat levels relative to wild type. Both results were confirmed by Sudan Black fixative-based staining (Figure S6). Reduced fat content of *dop-3* mutants was consistent with previous identification of this gene in a large-scale Nile Red based RNAi screen for fat regulatory genes [13]. The fat phenotype of *dop-1;dop-3* double mutants were similar to that of *dop-1* animals, suggesting that the *dop-1* function is epistatic to that of *dop-3* in regards to fat regulation. Interestingly, while *dop-2* mutants alone had wild type fat levels, *dop-1;dop-2* and *dop-2;dop-3* double mutants as well as a *dop-1;dop-2;dop-3* triple mutant and *dop-1;dop-2;dop-3;dop-4* quadruple mutant had nearly wild type fat levels. These findings suggest an intricate regulatory relationship among the receptors tested and that disruption of endogenous dopamine signaling influences worm's fat content as was observed in *dop-3* and *dop-1* mutants.

To verify whether exogenously supplied dopamine exerts its fat reducing effects through the identified receptors we exposed the mutant animals to dopamine (Figure 2 A and B). Relative to their base-line fat levels, only *dop-3* mutants appeared resistant to dopamine induced fat reduction. However, the low basal fat level of *dop-3* mutants complicated this interpretation. Various strains carrying combinations of mutated receptors were susceptible to the fat reducing effects of dopamine. By contrast, *dop-1;dop-4* double mutants and *dop-1;dop-2;dop-3;dop-4* quadruple mutants were partially and significantly resistant to the Nile Red fluorescence reducing effects of dopamine. This suggests that dopamine receptors are necessary for the fat reduction effect of exogenous dopamine. However, the exact mechanism is not completely elucidated given that a complete blockage of dopamine's induced fat reduction has not been achieved.

### Fat reducing effects of dopamine occur through elevated fat oxidation

Changes in fat levels ultimately indicate a change in the balance between caloric intake and energy expenditure. Dopamine may then diminish *C. elegans* fat reservoirs by reducing food ingestion or by increasing fat burn.

**Figure 3:**
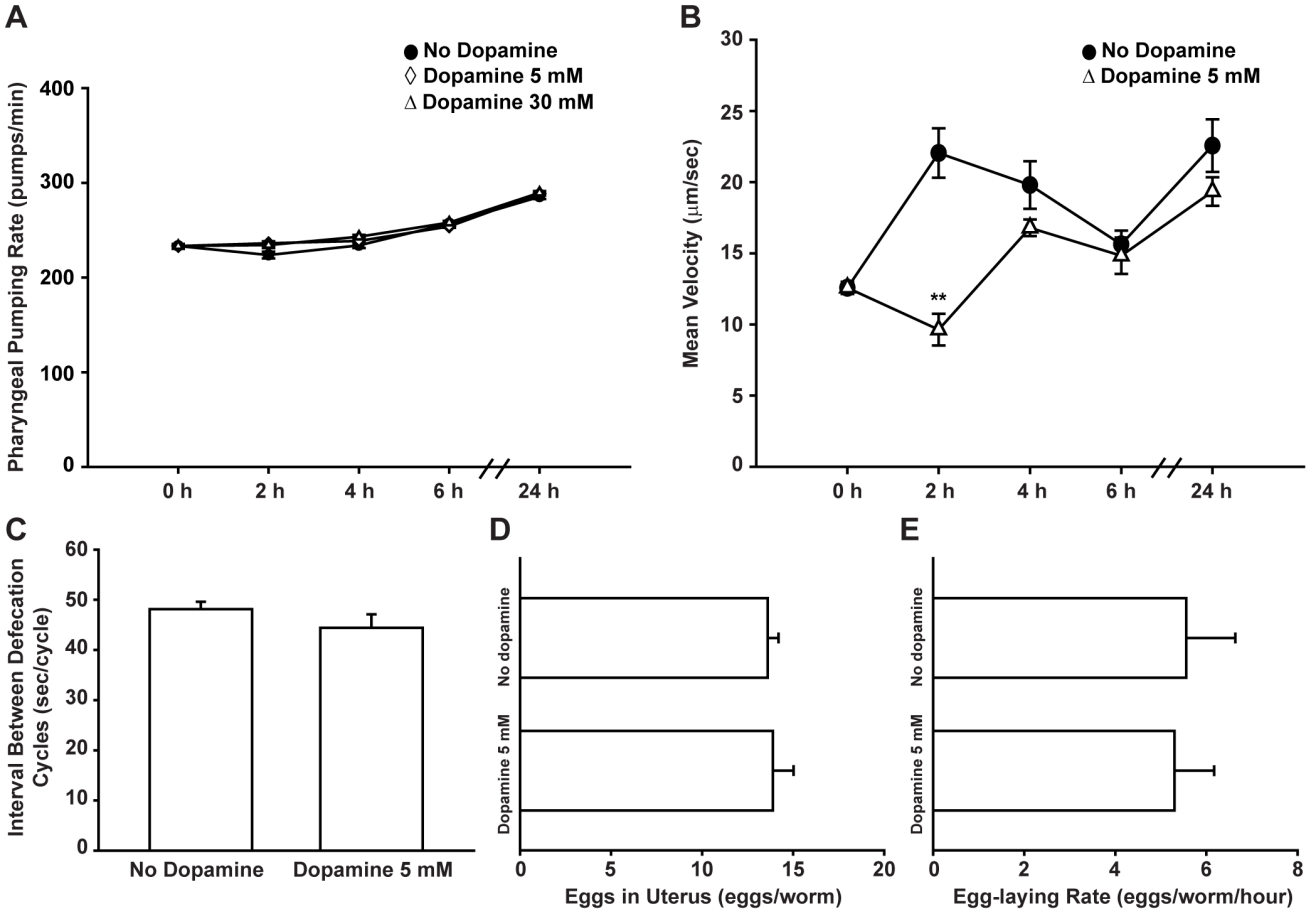
Fat reducing effect of dopamine treatment is not due to behavioral changes. (A) Pharyngeal pumping rate of wild type animals exposed to dopamine is similar to that of vehicle-treated animals over time. Data are presented as an average of 15 animals per condition per time ± SEM. (•) No dopamine, (◊) Dopamine 5 mM, (Δ) Dopamine 30 mM. (B) Dopamine causes a transient reduction in movement of wild type animals. Data are showed as the average of 5 to 9 animals per condition per time ± SEM. (•) No dopamine, (Δ) Dopamine 5 mM. ** p<0.01 compared to “No dopamine” control animals. (C) Defecation rate of dopamine-treated animals is similar to vehicle-treated animals. Data are presented as the average of 10 animals per condition ± SEM. (D–E) Chronic dopamine exposure does not affect progeny production in wild type animals. (D) Number of eggs in uterus or (E) laid per hour. Data are shown as average of nine animals per condition ± SEM.

*C. elegans* swallow nutrients through a pharynx pumping movement. The rate of pharyngeal pumping is modulated by food presence and quality, as well as by previous nutritional status and can be used as a surrogate for the amount of food intake [50] [51]. Despite the dramatic effects of dopamine on fat reduction, the pharyngeal pumping rate of wild type animals treated with dopamine were indistinguishable from those of mock-treated animals (Figure 3 A, Figure S2C). Defecation rate is another aspect related to food availability. At high concentrations dopamine has been reported to decrease defecation rate [52]. At the concentrations tested, defecation of gut lumen contents were similar between dopamine treated and control animals (Figure 3 C). Taken together, our results indicate that the fat reducing effects of dopamine cannot be attributed to significant changes in feeding rate or food access in gut lumen.

Studies have shown that high concentrations of dopamine decrease locomotion or even paralyze *C. elegans* [17] [20] [53]. A trivial explanation for the observed fat phenotype could be the inability of dopamine-treated animals to reach a nutrient source. To test this hypothesis we measured the movement of animals over time using an automated tracking system. While dopamine exposure caused an acute reduction in locomotion, this effect was transient and animals fully resumed a wild type phenotype within four hours despite continued treatment (Figure 3 B). It is of note that animals were kept in the presence of food at all times and exhibited wild type feeding rates as shown above. Dopamine's induced fat reduction was persistent and only reversed when animals were taken off treatment, a process that was not associated with any notable change in locomotion. Thus transient dopamine induced reduction in movement is unlikely to account for the dramatic and persistent fat reducing effects. Finally, as previously shown [53] [54], dopamine treatment did not alter progeny production rate, a central energy consuming event (Figure 3 D and E). Worth mentioning, exposed animals had no obvious developmental defects and appeared healthy. Taken together, our data indicate that dopamine induced fat reduction is unlikely to be a secondary consequence of increased rate of energy expenditure through locomotion, growth or reproduction.

**Figure 4:**
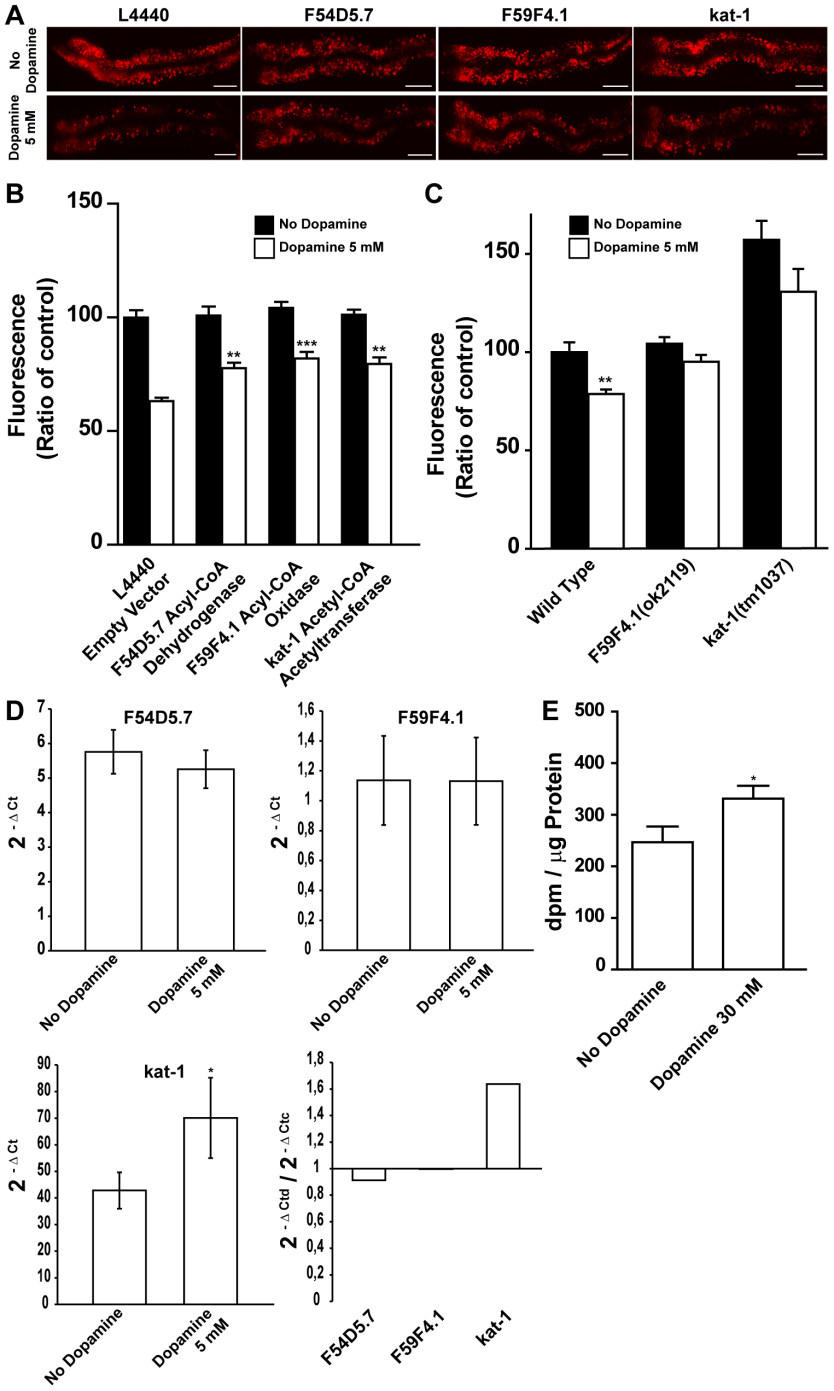
Dopamine induced fat reduction requires β-oxidation machinery. (A–B) Dopamine induced fat reduction is reduced by RNAi mediated knockdown of β-oxidation enzymes. (A) Representative images and (B) quantification of wild type animals fed with the indicated RNAi clones. Black and white bars represent vehicle and 5 mM dopamine treated animals, respectively. (C) Fluorescence quantification of 5 mM dopamine treated mutant worms available to corresponding RNAi gene clones. Data are showed as percentage of “No dopamine” control average ± SEM. (n = 8–10 animals per condition) (B) ** p<0.01 and *** p<0.001 compared to dopamine-treated animals fed on empty vector. (C) ** p<0.01 compared to “No dopamine” control animals of the same genotype. (D) Change in transcriptional levels of indicated metabolic genes upon 5 mM dopamine exposure quantified by qPCR. Dopamine increased kat-1 transcriptional level. Data are presented as average of three independent populations ± SD. Ctd – Ct dopamine exposed worms/Ctc – Ct No dopamine worms. * p<0.05 compared to “No dopamine” control animals. (E) Dopamine-treated animals exhibit an increase in β-oxidation rate. Data are presented as average of 12 independent populations ± SEM. * p<0.05 compared to “No dopamine” control animals.

We next sought to determine whether dopamine induced fat reduction is dependent on the activity of various metabolic pathways. To do so, the effects of RNAi induced silencing of 86 genes encoding known or predicted components of fat and sugar breakdown and synthesis pathways were examined in the dopamine induced fat reduction phenotype (Figure S7). We found and confirmed that RNAi-mediated inactivation of F54D5.7, predicted to encode an acyl-CoA dehydrogenase, F59F4.1, predicted to encode an acyl-CoA oxidase-1, and T02G8.5/*kat-1*, predicted to encode a ketoacyl-CoA thiolase, each partially but significantly abrogated the fat reducing effects of dopamine. A comparable outcome was observed when F59F4.1 and *kat-1* mutants were exposed to dopamine (Figure 4 A–C). F54D5.7 and F59F4.1 are predicted to encode enzymes that catalyze the first step of fat breakdown in mitochondria and peroxisomes, while KAT-1 thiolase is responsible for the release of acetyl-CoA at the final step of each β-oxidation cycle [55] [31]. Curiously, the knockdown of these genes or the loss-of-function mutation in F59F4.1 did not lead to fat accumulation as would be expected assuming that they are β-oxidation enzymes. We hypothesized that other genes coding for isoenzymes may have their transcriptional level changed to maintain energetic equilibrium although dopamine requires them to exert its effect on fat stores. To assess whether dopamine modulates gene expression we measured transcriptional levels through qPCR. We found that dopamine increased expression of *kat-1* (Figure 4 D). Of note *kat-1* expression is reduced in animals exposed to serotonin [15]. These results indicate that dopamine induced fat reduction may result from changes in the fat oxidation rate. To directly test this hypothesis we fed dopamine exposed *C. elegans* radio-labeled oleic acid and measured the production of radio-labeled water as an indicator of the rate of β-oxidation (Figure 4 E). 30 mM dopamine concentration was chosen because it results in a strong fat reduction. Consistent with the genetic results, worms exposed to dopamine for 24 hours had an increased level of fat oxidation. Together these findings are consistent with a mechanism where dopamine causes fat reduction by increasing flux of fatty acids through oxidative pathways.

## Discussion

In this report we sought to determine the relationship between dopamine and fat in the nematode *C. elegans*. We observed that animals exposed to dopamine exhibited reduced fat storage over time. Our results suggest that the fat reduction phenotype is not an indirect outcome of other dopamine modulated behavior but rather an independent response. Therefore, we attempted to identify components of the dopaminergic system linked to fat metabolism and found an intricate interaction between dopamine receptors. It appears that dopamine receptors DOP-1 and DOP-4 signal in cascades important for mobilization of lipid stores while DOP-2 and DOP-3 somehow modulate the process. The finding that treatment with supraphysiological levels of exogenous dopamine caused reduced fat storage was intriguing, but by itself did not prove that dopamine signaling regulates fat storage. At high concentrations it is possible that dopamine affects other amine signaling pathways, or other pathways independent of the endogenous dopaminergic pathway. However, individual dopamine receptors DOP-1 and DOP-3 have their own fat storage phenotypes and the magnitude of fat reduction induced by exogenous dopamine was significantly smaller in *dop-1;dop-2;dop-3;dop-4* quadruple mutants. Although smaller, *dop-1;dop-2;dop-3;dop-4* quadruple mutants response to dopamine suggest that other dopamine receptors may be present in *C. elegans*. In fact, studies have proposed this already [20], [44]. From the seventeen putative biogenic amine receptors, at least three remain without specific ligand characterization. Further, MOD-1 mutants are fairly responsive to serotonin induced fat reduction although they show a similarly wild type response to dopamine. These results support a specific role for the endogenous dopamine signaling in fat metabolism regulation.

As in mammals, the *C. elegans* nervous system regulates fat storage through the control of feeding and energy expenditure [39]. As expected for a critical physiological process, this control is complex and involves several neuroendocrine pathways [13] [16] [56] [57]. For instance, while serotonin signaling in *C. elegans* regulates both fat and feeding, it does so through distinct molecular pathways, and fat reducing effects are ultimately due to increased peripheral fat oxidation [45] [35]. Given that both serotoninergic and dopaminergic systems are activated when *C. elegans* encounters food, their straight regulation of energy metabolism is an elegant way to integrate new food sources and energy reserves, thereby releasing products for costly energy behaviors like reproduction. Curiously, food availability has a major influence on worm's reproduction. Therefore independent neural regulation of feeding behavior and metabolism appears to be a general response elicited by the perception of food. These findings should motivate the search for mechanisms that link signaling through neurally expressed dopaminergic receptors and fat mobilization.

Compelling data suggest a role for dopamine signaling on weight control. Murine studies have highlighted the motivational aspect of dopamine that drives feeding and the disruption of the dopaminergic system has been associated with overconsumption of food in obese rats [58] [59]. In humans, reduced dopamine signaling, whether through pharmacological manipulation or through examination of receptor density, is associated with obesity [11] [12] [60] [61]. Nevertheless, the precise interplay of dopamine in fat regulatory mechanisms has remained elusive. In *C. elegans*, we observed that dopamine induces changes in fat storage though modifications in peripheral fat oxidation pathways. Fat breakdown is orchestrated by many genes, some of which functioning at the same biochemical step. Therefore changes in transcriptional levels of genes encoding isoenzymes may result in similar phenotypes. *C. elegans* genome encodes at least seven acyl-CoA oxidases according to wormbase.org and counter-regulation of these genes may explain the wild type fat phenotype observed on F59F4.1 mutants. The idea that dopamine may influence fat reserves through modulation of cellular bioenergetics brings forth a new aspect of dopamine signaling to be explored. Indeed, treatment with a dopamine D2 receptor agonist has recently been found to increase resting energy expenditure in women [62] [63]. Therefore, as in *C. elegans*, the ancient role of dopamine signaling in the regulation of peripheral metabolism through specific pathways may be conserved in humans.

## Supporting Information

Figure S1 **Dopamine induced fat reduction requires continued exposure to dopamine.** (A–B) Dopamine induced fat reduction requires continues exposure to the neurotransmitter. (A) Diagram of the experiment with representative images taken for each condition. (B) Fluorescence quantification shows that transfer of dopamine exposed wild type animals to plates without dopamine brings fat stores up to non-treated animal levels. Data are expressed as percentage of 24 h vehicle-treated animals average ± SEM. (n = 8–10 animals per condition). *** p<0.001 compared to vehicle-treated animals for each time point. (C) Fat reduction induced by dopamine and serotonin (5-HT). Quantification of fluorescence of animals exposed to dopamine and/or serotonin. Data are expressed as percentage of 24 h vehicle-treated animals average ± SEM. (n = 8–10 animals per condition). *** p<0.001 compared to vehicle-treated animals, ### p<0.001 compared to dopamine-treated animals, ns means “not significant”. (D) Increasing dopamine's concentrations reduce Nile Red fluorescence similarly in wild type and *mod-1* serotonin receptor mutant animals. Black and white bars represent wild type and *mod-1* animals respectively. Data are expressed as percentage of vehicle-treated worms mean for each genotype ± SEM. (n = 8–10 animals per condition per genotype). * p<0.05, ** p<0.01, *** p<0.001 compared to vehicle-treated animals of the same genotype.
(TIF)

Figure S2 **Dopamine fat reducing effect is dose dependent.** (A–B) Fat stores are reduced in dopamine-exposed animals as visualized by vital dye Nile Red. (A) Fluorescence quantification of animals exposed to a smaller concentration range of dopamine. Box and whiskers represent 25–75^th^ percentile with mean and minimum to maximum measured values, respectively. Data are shown as percentage of “No dopamine” control. (n = 20 animals per condition). (B) Dopamine inhibits accumulation of fat over time. (•) No dopamine, (Δ) Dopamine 5 mM. Data are shown as percentage of 0 hour “No dopamine” animals average ± SEM. (n = 15–20 animals per condition per time) ** p<0.01 and *** p<0.001 compared to “No dopamine” control animals. (C) Pharyngeal pumping of wild type and dopamine receptor loss-of-function mutants is similar. Black and white bars represent vehicle and 5 mM dopamine treated animals, respectively. Data are presented as percentage of vehicle-treated wild-type average ± SEM. (n = 10 animals per condition per genotype).
(TIF)

Figure S3 **A new protocol for use of vital dye Nile Red highlights different tissues lipid droplets.** (A) LipidTOX is a triglyceride fixative-based dye. The figure shows worm's diverse lipid storage tissues. White arrow highlights yolk stained in gonads. These are representative images from three different experiments. DIC means differential interference contrast microscopy. (B) Acute instead of chronic exposure to Nile Red reveals lipid droplets outside intestinal cells. The right top panel shows Nile Red staining of gonad (white arrow), intestine and hypodermis. To confirm that stained structures are mainly lipid droplets, a transgenic strain (VS20) carrying adipose triglyceride lipase-1 tagged with green fluorescent protein (ATGL-1::GFP) was used. The bottom panel shows Nile Red staining of VS20. ATGL-1::GFP colocalizes with Nile Red. Representative images of different animals from the same experiment.
(TIF)

Figure S4 **Worm's developmental stage affects lipid profile as assessed by thin-layer chromatography (TLC) from whole animal homogenates.** (A) Representative images of TLC plates loaded with extracts from different developmental stages animals. Seq1 to Seq4 are distinct chromatograms. Some extracts were loaded on more than one plate with similar results. (B) Quantification of lipid fractions from each population. Graph on the left shows absolute quantification. Graph on the right shows corresponding percentage of each lipid fraction in total lipid extract for each group. Bar colors in (B) correspond to the bands in (A). Data are presented as a mean of three biological replicates extracted from 10000 worms each ± SD. L2 – second larval molt. L2/L3 – L2 to L3 molt. L4 – fourth larval molt. L4/YA – L4 to young adults (YA) molt. GA – Gravid adults.
(TIF)

Figure S5 **Dopamine does not change expression of serotonin regulated metabolic genes.** (A–B) Quantitative real-time polymerase chain reaction measurement of transcriptional levels of specified metabolic genes after exposure to different dopamine concentrations. (A) Graphs show mean transcriptional levels of three independent populations ± SD. Data points were calculated using the follow equation: 2 ^−Δ Ct^, where −Δ Ct = −(Ct dopamine treated or “No dopamine” control animals – Ct geometric mean of housekeeping genes of the same sample) and Ct =  cycle threshold calculated by the qPCR software (ABI 7500). Black and white bars represent vehicle and dopamine treated animals, respectively. (B) 2^−Δ Ctd^/2^−Δ Ctc^ ratio indicates fold changes in gene expression after exposure to dopamine. Ctd =  cycle threshold of dopamine exposed animals and Ctc =  cycle threshold of “No dopamine” control animals.
(TIF)

Figure S6 **Sudan Black staining of dopamine receptor loss-of-function mutants.** (A–C) *dop-1* and *dop-3* mutants have increased and decreased fat stores, respectively. (A) *dop-1* and (B) *dop-3* representative images of fixed-animals stained with Sudan Black. Images of wild type animals stained in the same tube are shown. (C) quantitation of Sudan Black stain intensities. Data are expressed as percentage of wild type animals average ± SD. (n = 5–8 animals per condition). * p<0.05 compared to wild type animals.
(TIF)

Figure S7 **Compilation of the dopamine metabolic screen.** Nile Red fluorescence quantifications of worms fed on a series of RNAi clones are shown. Black and white bars represent vehicle and 5 mM dopamine-treated animals, respectively. Clones that suppressed dopamine fat reduction phenotype more than 80% of the “No dopamine” control's reduction are highlighted in red and were picked for further analyses. Data are presented as fluorescence arbitrary units (a.u.) ± SEM (n = 5–40 animals per condition).
(TIF)

Table S1 **List of primers and cycle thresholds (Ct) used for gene transcriptional level measurements.**
(XLSX)
