# Supplementary figures and images for: Dopamine Signaling Regulates Fat Content through β-Oxidation in Caenorhabditis elegans
Source: PLoS One. 2014 Jan 22;9(1):e85874. doi: 10.1371/journal.pone.0085874 (PMC3899111; doi:10.1371/journal.pone.0085874)

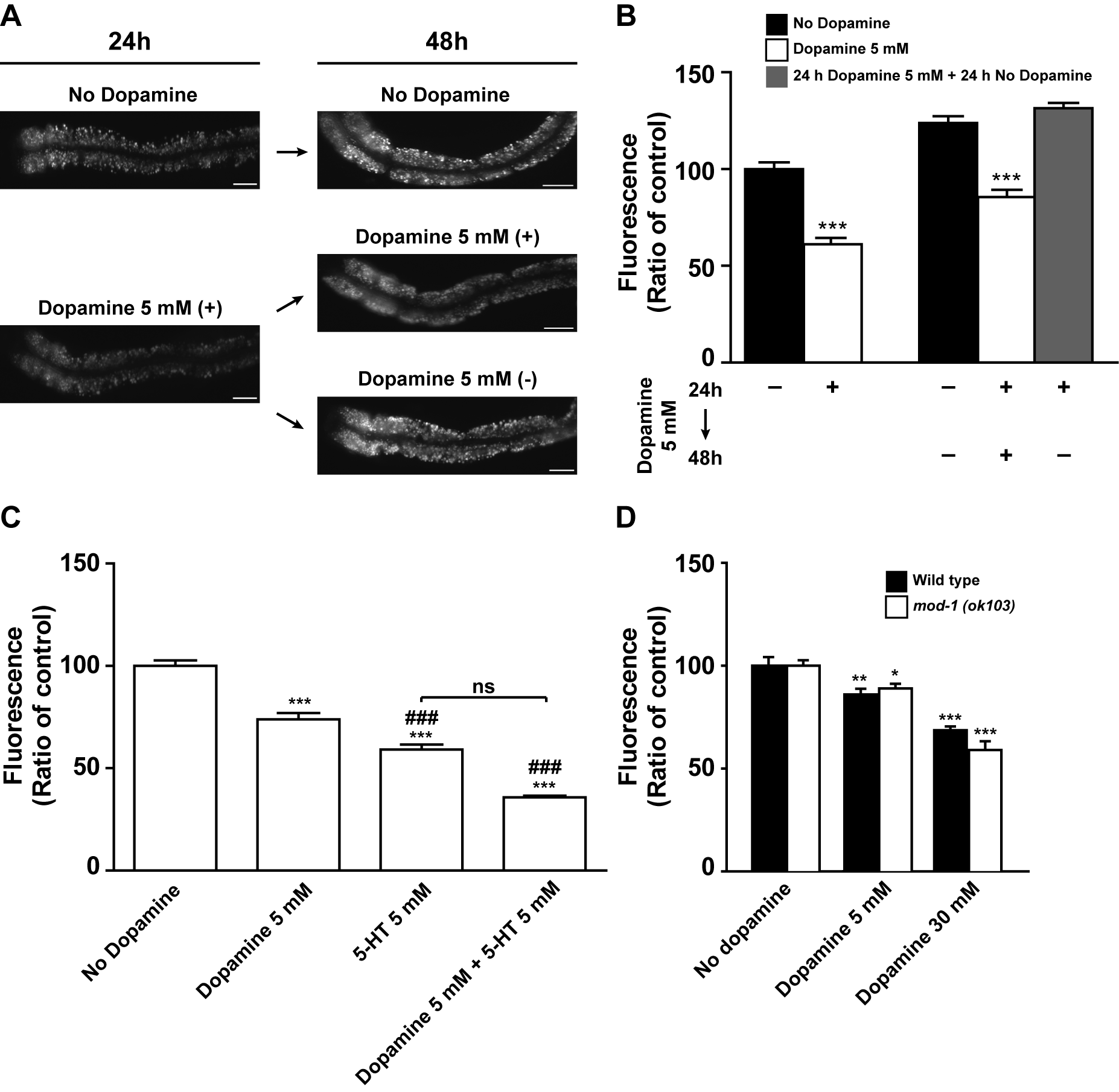

Supplement: Figure S1 — Dopamine induced fat reduction requires continued exposure to dopamine. (A–B) Dopamine induced fat reduction requires continues exposure to the neurotransmitter. (A) Diagram of the experiment with representative images taken for each condition. (B) Fluorescence quantification shows that transfer of dopamine exposed wild type animals to plates without dopamine brings fat stores up to non-treated animal levels. Data are expressed as percentage of 24 h vehicle-treated animals average ± SEM. (n = 8–10 animals per condition). *** p<0.001 compared to vehicle-treated animals for each time point. (C) Fat reduction induced by dopamine and serotonin (5-HT). Quantification of fluorescence of animals exposed to dopamine and/or serotonin. Data are expressed as percentage of 24 h vehicle-treated animals average ± SEM. (n = 8–10 animals per condition). *** p<0.001 compared to vehicle-treated animals, ### p<0.001 compared to dopamine-treated animals, ns means “not significant”. (D) Increasing dopamine's concentrations reduce Nile Red fluorescence similarly in wild type and mod-1 serotonin receptor mutant animals. Black and white bars represent wild type and mod-1 animals respectively. Data are expressed as percentage of vehicle-treated worms mean for each genotype ± SEM. (n = 8–10 animals per condition per genotype). * p<0.05, ** p<0.01, *** p<0.001 compared to vehicle-treated animals of the same genotype. (TIF) [file pone.0085874.s001.tif]

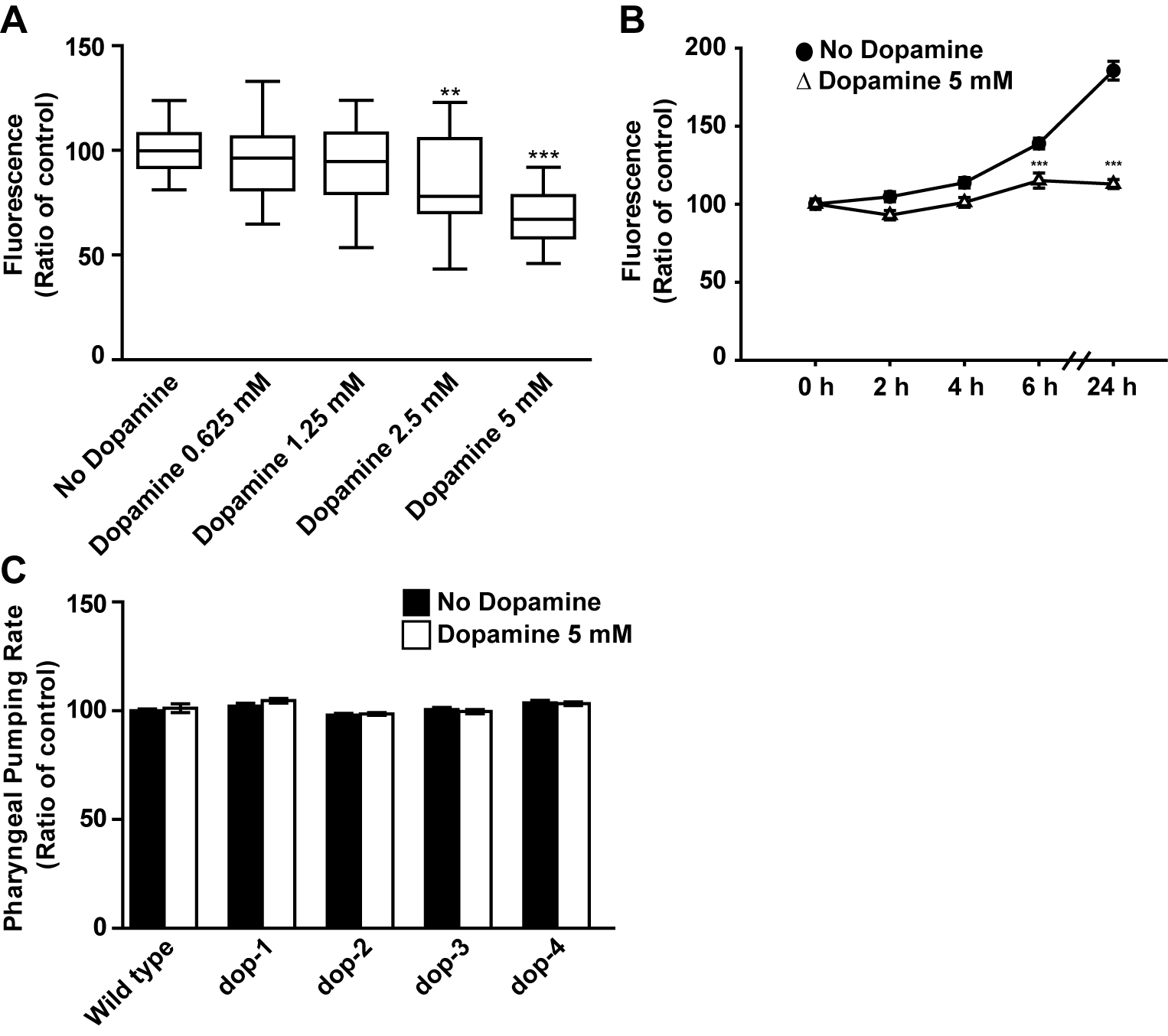

Supplement: Figure S2 — Dopamine fat reducing effect is dose dependent. (A–B) Fat stores are reduced in dopamine-exposed animals as visualized by vital dye Nile Red. (A) Fluorescence quantification of animals exposed to a smaller concentration range of dopamine. Box and whiskers represent 25–75th percentile with mean and minimum to maximum measured values, respectively. Data are shown as percentage of “No dopamine” control. (n = 20 animals per condition). (B) Dopamine inhibits accumulation of fat over time. (•) No dopamine, (Δ) Dopamine 5 mM. Data are shown as percentage of 0 hour “No dopamine” animals average ± SEM. (n = 15–20 animals per condition per time) ** p<0.01 and *** p<0.001 compared to “No dopamine” control animals. (C) Pharyngeal pumping of wild type and dopamine receptor loss-of-function mutants is similar. Black and white bars represent vehicle and 5 mM dopamine treated animals, respectively. Data are presented as percentage of vehicle-treated wild-type average ± SEM. (n = 10 animals per condition per genotype). (TIF) [file pone.0085874.s002.tif]

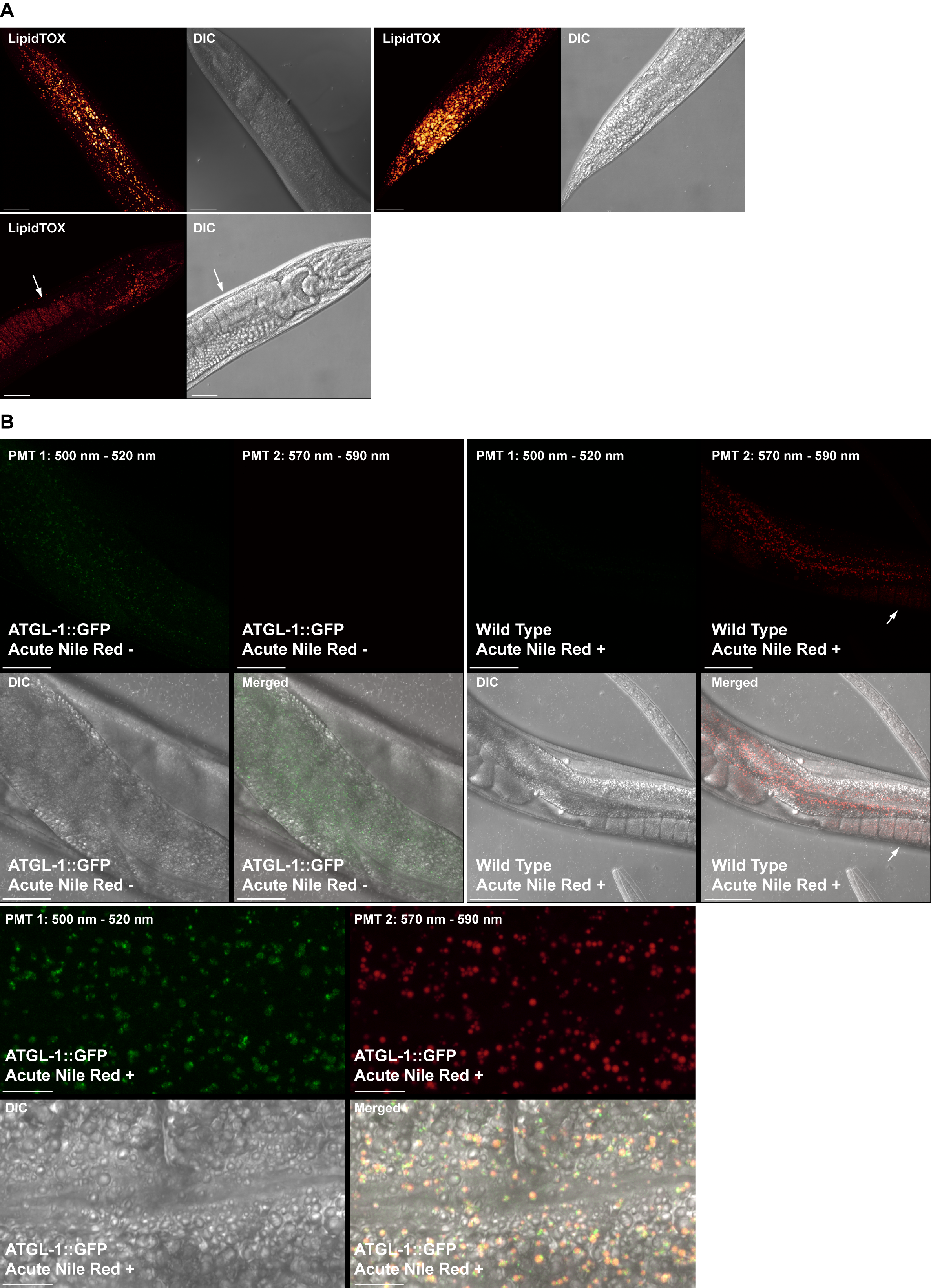

Supplement: Figure S3 — A new protocol for use of vital dye Nile Red highlights different tissues lipid droplets. (A) LipidTOX is a triglyceride fixative-based dye. The figure shows worm's diverse lipid storage tissues. White arrow highlights yolk stained in gonads. These are representative images from three different experiments. DIC means differential interference contrast microscopy. (B) Acute instead of chronic exposure to Nile Red reveals lipid droplets outside intestinal cells. The right top panel shows Nile Red staining of gonad (white arrow), intestine and hypodermis. To confirm that stained structures are mainly lipid droplets, a transgenic strain (VS20) carrying adipose triglyceride lipase-1 tagged with green fluorescent protein (ATGL-1::GFP) was used. The bottom panel shows Nile Red staining of VS20. ATGL-1::GFP colocalizes with Nile Red. Representative images of different animals from the same experiment. (TIF) [file pone.0085874.s003.tif]

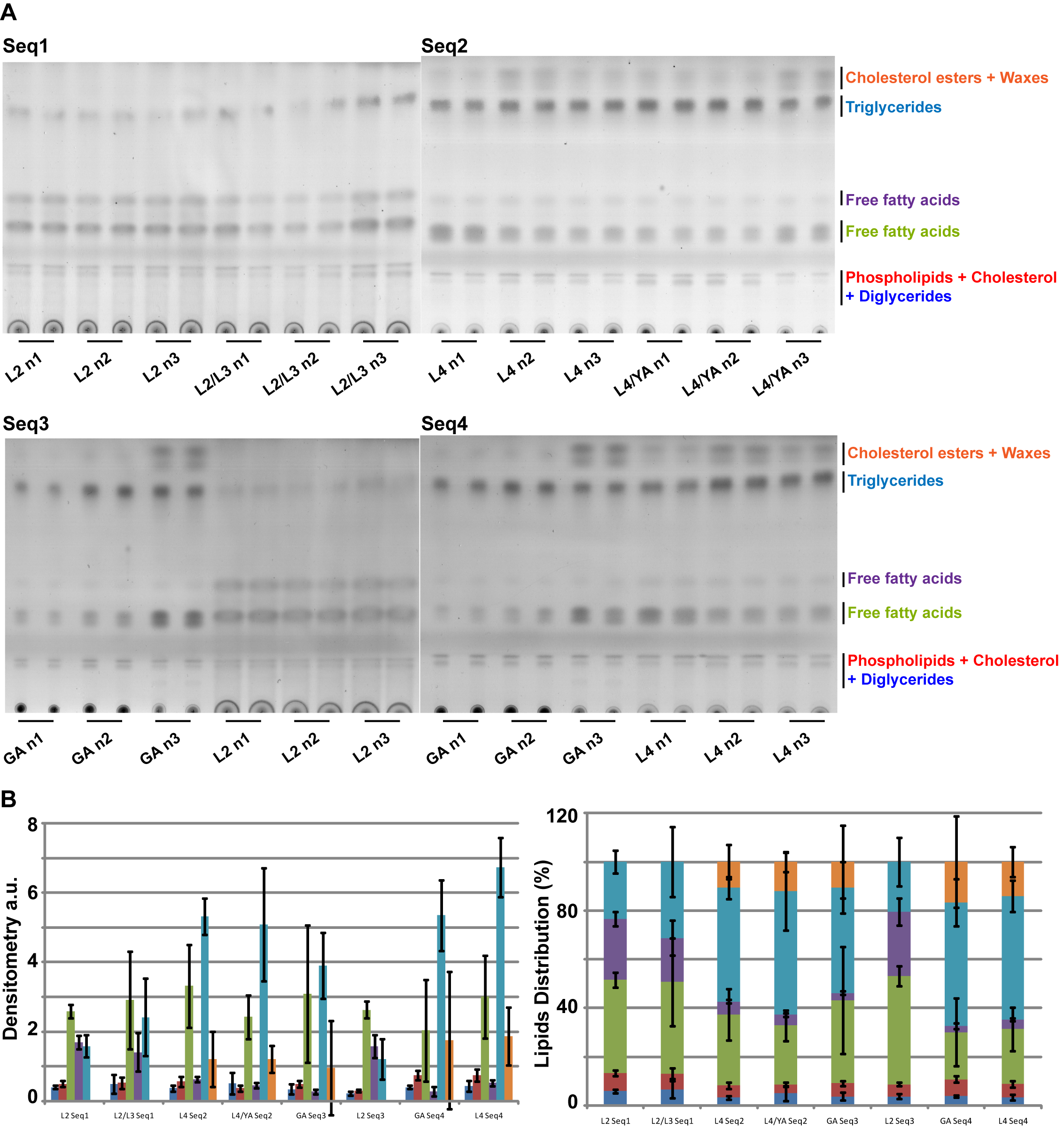

Supplement: Figure S4 — Worm's developmental stage affects lipid profile as assessed by thin-layer chromatography (TLC) from whole animal homogenates. (A) Representative images of TLC plates loaded with extracts from different developmental stages animals. Seq1 to Seq4 are distinct chromatograms. Some extracts were loaded on more than one plate with similar results. (B) Quantification of lipid fractions from each population. Graph on the left shows absolute quantification. Graph on the right shows corresponding percentage of each lipid fraction in total lipid extract for each group. Bar colors in (B) correspond to the bands in (A). Data are presented as a mean of three biological replicates extracted from 10000 worms each ± SD. L2 – second larval molt. L2/L3 – L2 to L3 molt. L4 – fourth larval molt. L4/YA – L4 to young adults (YA) molt. GA – Gravid adults. (TIF) [file pone.0085874.s004.tif]

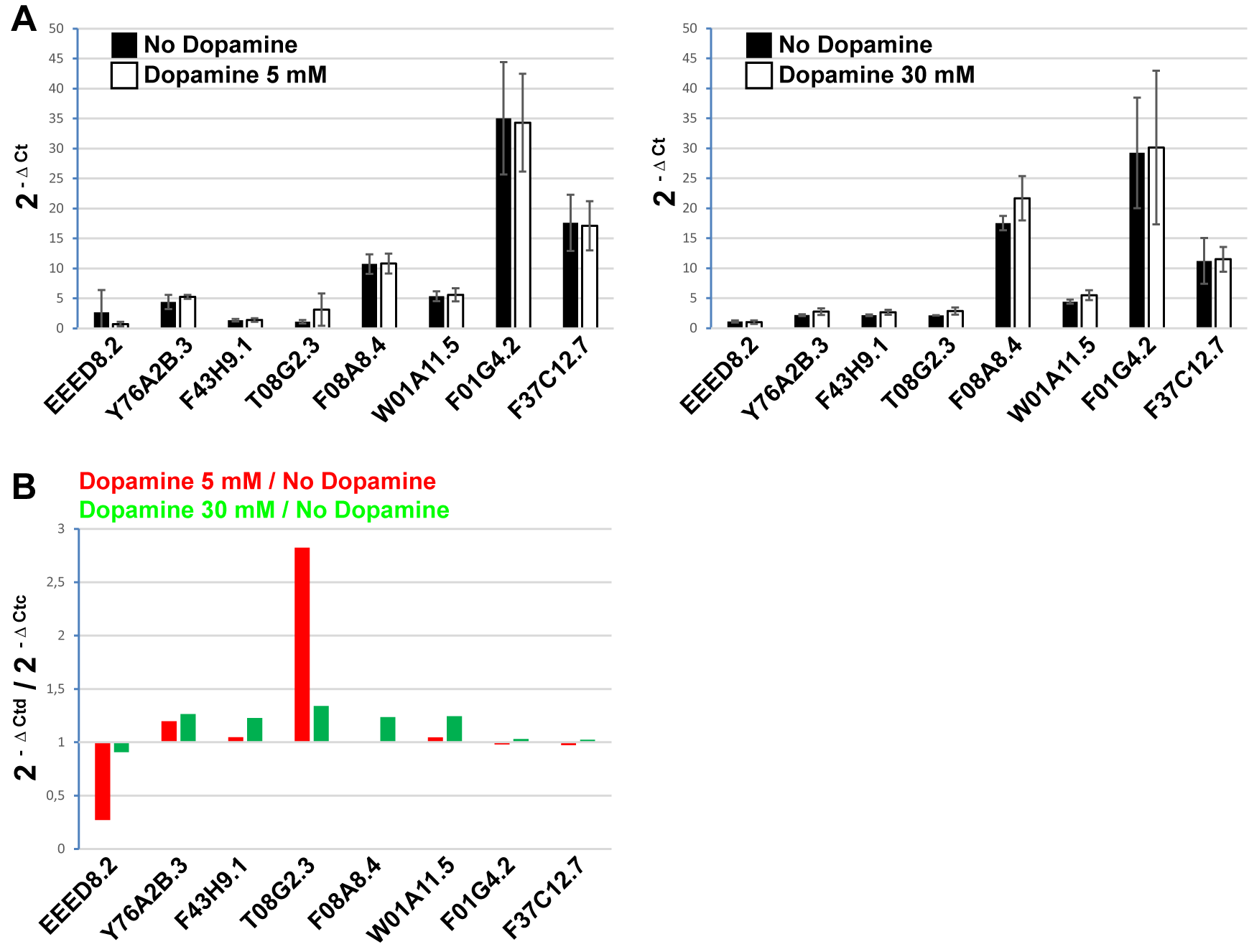

Supplement: Figure S5 — Dopamine does not change expression of serotonin regulated metabolic genes. (A–B) Quantitative real-time polymerase chain reaction measurement of transcriptional levels of specified metabolic genes after exposure to different dopamine concentrations. (A) Graphs show mean transcriptional levels of three independent populations ± SD. Data points were calculated using the follow equation: 2 −Δ Ct, where −Δ Ct = −(Ct dopamine treated or “No dopamine” control animals – Ct geometric mean of housekeeping genes of the same sample) and Ct = cycle threshold calculated by the qPCR software (ABI 7500). Black and white bars represent vehicle and dopamine treated animals, respectively. (B) 2−Δ Ctd/2−Δ Ctc ratio indicates fold changes in gene expression after exposure to dopamine. Ctd = cycle threshold of dopamine exposed animals and Ctc = cycle threshold of “No dopamine” control animals. (TIF) [file pone.0085874.s005.tif]

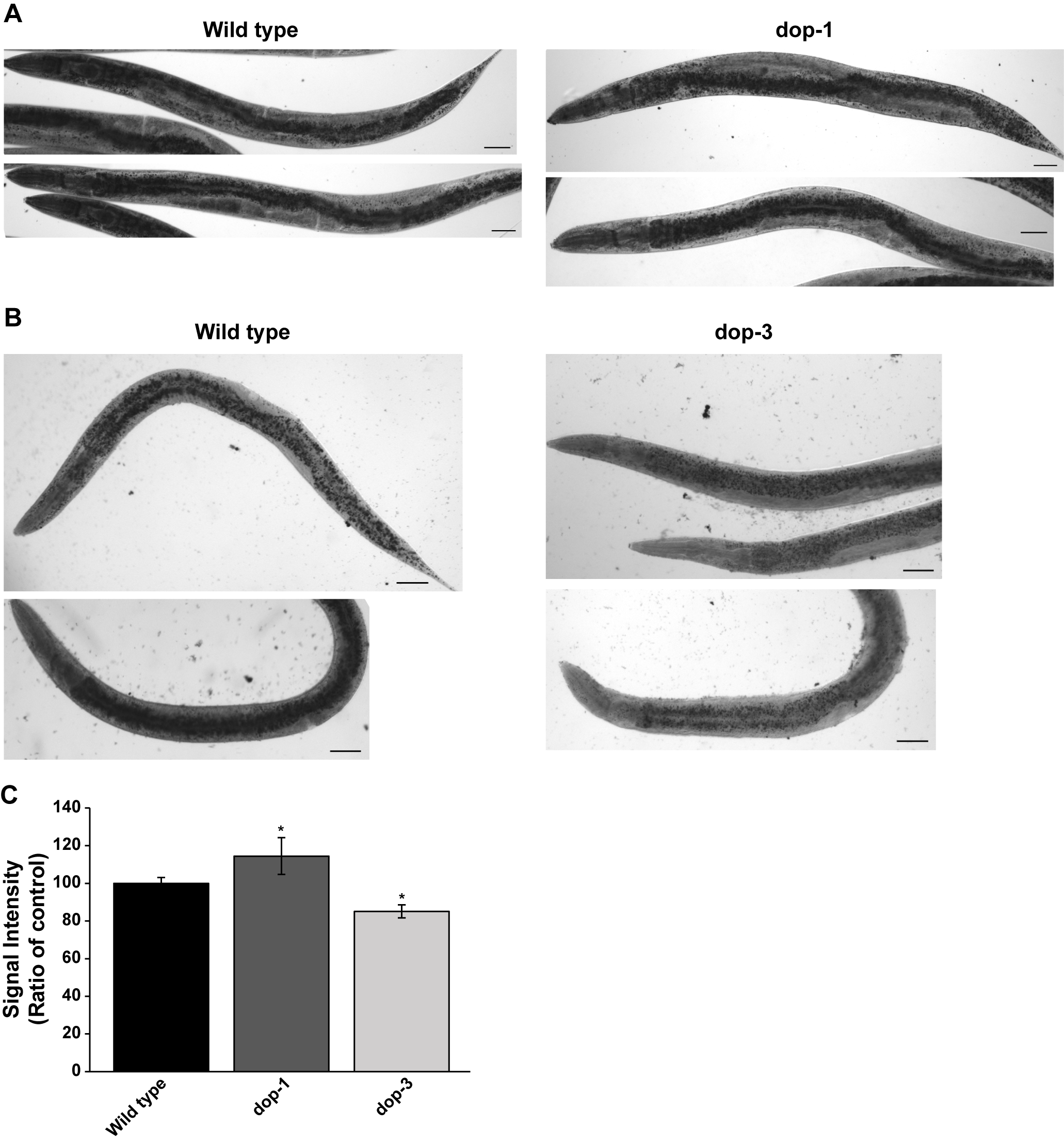

Supplement: Figure S6 — Sudan Black staining of dopamine receptor loss-of-function mutants. (A–C) dop-1 and dop-3 mutants have increased and decreased fat stores, respectively. (A) dop-1 and (B) dop-3 representative images of fixed-animals stained with Sudan Black. Images of wild type animals stained in the same tube are shown. (C) quantitation of Sudan Black stain intensities. Data are expressed as percentage of wild type animals average ± SD. (n = 5–8 animals per condition). * p<0.05 compared to wild type animals. (TIF) [file pone.0085874.s006.tif]

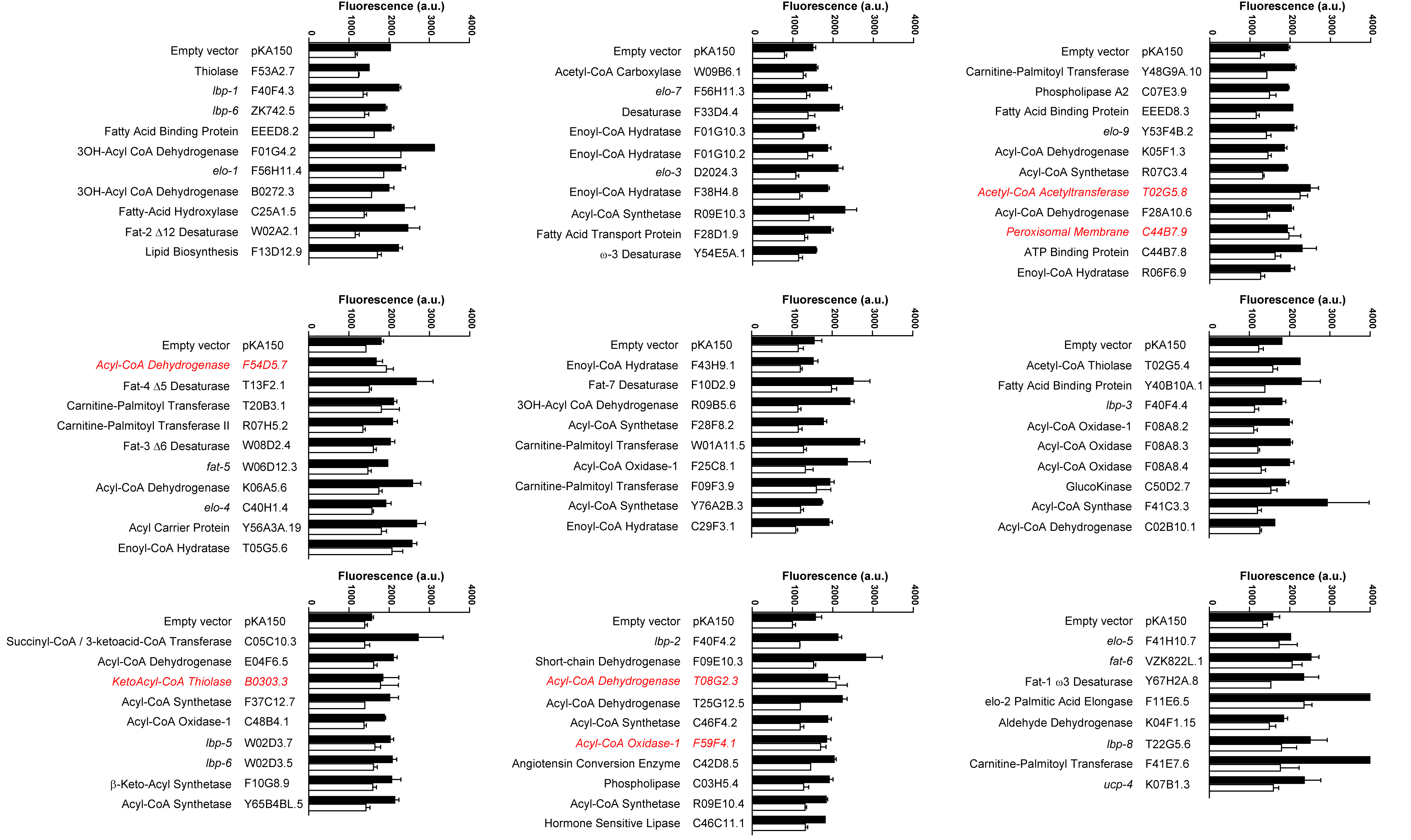

Supplement: Figure S7 — Compilation of the dopamine metabolic screen. Nile Red fluorescence quantifications of worms fed on a series of RNAi clones are shown. Black and white bars represent vehicle and 5 mM dopamine-treated animals, respectively. Clones that suppressed dopamine fat reduction phenotype more than 80% of the “No dopamine” control's reduction are highlighted in red and were picked for further analyses. Data are presented as fluorescence arbitrary units (a.u.) ± SEM (n = 5–40 animals per condition). (TIF) [file pone.0085874.s007.tif]
